# The Effectiveness Of Digital Interventions To Increase Preventive Care Uptake In Older Adults: A Systematic Review

**DOI:** 10.1093/geroni/igaf122.3346

**Published:** 2025-12-31

**Authors:** Lindsay Burton, Kathy Rush, Mindy A Smith, Robert Janke

**Affiliations:** The University of British Columbia, Vancouver, British Columbia, Canada; The University of British Columbia - Okanagan Campus, Kelowna, British Columbia, Canada; The University of British Columbia, Vancouver, British Columbia, Canada; The University of British Columbia, Vancouver, British Columbia, Canada

## Abstract

The coming decade will witness a substantial increase in older adult populations, contributing to a surge in healthcare utilization and costs driven by rising chronic diseases. Addressing these challenges necessitates strategies to promote healthy aging, reduce chronic diseases, and enhance quality of life among older adults. One proactive approach is to encourage older individuals to follow recommended preventive care and immunization schedules. Despite the increasing number of recommended immunizations for older adults, completion rates remain alarmingly low, particularly for pneumococcal and shingles vaccines. What is unknown is how the use of digital interventions can be appropriately leveraged to address this gap in older adult health services utilization. Following PRISMA guidelines, this systematic review aimed to assess the range and effectiveness of digital interventions to improve preventive uptake among older adults. Twenty-seven studies (23 randomized clinical trials and 4 quasi-experimental) were included. The preventive care services targeted in interventions included colorectal cancer screening (n = 14), breast cancer screening (n = 2), influenza vaccination (n = 5), combined vaccinations (n = 2), pneumococcal vaccination (n = 2), herpes zoster vaccination (n = 1), and COVID-19 vaccination (n = 1). Interventions primarily leveraged automated digital communication, combined digital and printed outreach, tailored telephone communication, and supplemented in-person promotion. Studies consistently demonstrated an increase in preventive care uptake post intervention. However, including digital elements did not consistently show a significant improvement over control groups or standard care. These findings emphasize the importance and effectiveness of a combination of approaches in enhancing preventive care uptake among older adults, thus contributing to improved public health outcomes in aging populations.

